# Predictive value of peripheral blood cell-free DNA breast cancer gene mutation profiling for postoperative pathological malignancy in BI-RADS 4 breast nodules

**DOI:** 10.3389/fgene.2026.1828106

**Published:** 2026-07-22

**Authors:** Guan Lyu, Huimin Duan, Xiaomei Sheng, Liangquan Liu, Jing Luo

**Affiliations:** 1 Department of Breast and Thyroid Surgery, Nanjing Gaochun People’s Hospital, Nanjing, Jiangsu, China; 2 Department of General Surgery, Ezhou Central Hospital, Ezhou, Hubei, China; 3 Department of Warehouse Management, Jinling Hospital, Medical School of Nanjing University, Nanjing, Jiangsu, China; 4 Department of Breast Surgery, Sichuan Provincial People’s Hospital, Chengdu, Sichuan, China

**Keywords:** BI-RADS 4, breast neoplasms, gene mutation profiling, liquid biopsy, peripheral blood cfDNA, prediction model

## Abstract

**Objective:**

To investigate the predictive value of peripheral blood cfDNA breast cancer gene mutation profiling for postoperative pathological malignancy in BI-RADS 4 breast nodules.

**Methods:**

Clinical data from 212 patients with BI-RADS 4 breast nodules at our hospital from January 2020 to January 2024 were retrospectively collected. Patients were divided into benign group (128 cases) and malignant group (84 cases) according to postoperative pathological results. Peripheral venous blood was collected within 1 week before surgery, cfDNA was extracted and targeted next-generation sequencing (NGS, coverage depth 500–1,000×) of 49 breast cancer-related genes was performed. cfDNA concentration, tumor mutation burden (TMB), mutation allele frequency (MAF), gene-specific mutation detection rates (BRCA1, TP53, ESR1, ERBB2, PIK3CA, *etc.*), and a composite Gene Mutation Score (GMS) were analyzed. Multivariate logistic regression analysis was used to identify independent predictors of postoperative pathological malignancy, and ROC curves were plotted to evaluate predictive efficacy.

**Results:**

The malignant group had higher cfDNA concentration (14.3 ± 5.8 vs. 8.6 ± 3.2 ng/mL, P < 0.001) and TMB [2.86 (1.54,4.72) vs. 0.42 (0.28,0.68) mut/Mb, P < 0.001] than the benign group, while overall mutation detection rate (2.18% ± 0.63% vs. 2.84% ± 0.52%, P < 0.001), mutation coverage in promoter/regulatory regions of key driver genes (36.2 ± 9.5 vs. 45.6 ± 8.3 RPK, P < 0.001), and GMS (0.95 ± 0.74 vs. 1.82 ± 0.68, P < 0.001) were lower in the malignant group, indicating distinct mutation patterns. Mutation rates in BRCA1, TP53, ESR1, and ERBB2 in the malignant group were significantly higher than those in the benign group (P < 0.001). G3 grade patients had higher TMB and lower GMS, BRCA1/TP53 MAF, and regulatory region coverage than G1/G2 grade patients (P < 0.05). Multivariate logistic regression analysis showed that age (OR = 1.058, 95%CI: 1.021–1.096), BI-RADS classification (OR = 2.874, 95%CI: 1.643–5.027), TMB (OR = 4.326, 95%CI: 2.214–8.452), and GMS (OR = 0.178, 95%CI: 0.082–0.388) were independent predictors of postoperative pathological malignancy (P < 0.05). The combined model (age + BI-RADS classification + TMB + GMS) had an AUC of 0.923 (95%CI: 0.884–0.962), sensitivity of 88.1%, specificity of 89.1%, positive predictive value of 84.1%, and negative predictive value of 91.8%, superior to single indicators (P < 0.001).

**Conclusion:**

cfDNA breast cancer gene mutation profiling has good predictive value for postoperative pathological malignancy in BI-RADS 4 breast nodules, with TMB and GMS being independent predictors. The combined model integrating clinical, imaging, and gene mutation features demonstrates excellent predictive efficacy and can provide a non-invasive, accurate assessment tool for preoperative risk stratification of BI-RADS 4 lesions.

## 1 Introduction

Breast cancer is the most common malignant tumor in women worldwide, seriously threatening women’s health (Xu et al., 2023). The Breast Imaging Reporting and Data System (BI-RADS) classification is an important tool for clinical assessment of breast lesion nature. BI-RADS 4 lesions have a malignancy risk of 2%–95%, with high heterogeneity, posing challenges to clinical decision-making (Yang et al., 2025). Currently, for BI-RADS 4 nodules, diagnosis mainly relies on biopsy for definitive diagnosis, but there are issues such as invasiveness, sampling limitations, and poor patient compliance. Therefore, there is an urgent need to develop efficient, non-invasive prediction methods to optimize clinical management strategies.

Cell-free DNA (cfDNA) refers to extracellular DNA fragments circulating in peripheral blood; a tumor-derived subset of cfDNA, carrying somatic mutations characteristic of the primary tumor, is specifically termed circulating tumor DNA (ctDNA). Throughout this study, the term cfDNA is used consistently to denote total circulating cell-free DNA extracted from plasma, which includes both tumor-derived and non-tumor-derived fractions. cfDNA has emerged as a promising liquid biopsy biomarker with great potential in early tumor diagnosis and monitoring (Liu and Wang, 2022). The release of cfDNA into plasma mainly occurs through pathways such as apoptosis and necrosis, and tumor-derived cfDNA carries somatic mutations characteristic of the primary tumor. Recent studies have found that mutations in breast cancer driver genes—including BRCA1, BRCA2, TP53, PIK3CA, ERBB2, and ESR1—can be detected in peripheral blood cfDNA even at early stages, reflecting the genetic landscape of the tumor (Penny et al., 2024; Doebley et al., 2022). These genomic alterations can serve as molecular markers for tumor diagnosis and prognosis. However, there are still few studies on the systematic application of cfDNA breast cancer gene mutation profiling in early diagnosis, particularly the predictive value for BI-RADS 4 clinical gray-zone lesions remains unclear (Barbirou et al., 2022). In addition, previous studies mostly focused on single cfDNA indicators such as concentration or fragment length, lacking systematic evaluation of multi-gene mutation patterns, and insufficient in-depth analysis of associations with clinicopathological features (Cao et al., 2024).

Therefore, this study systematically analyzes cfDNA breast cancer gene mutation features in patients with BI-RADS 4 breast nodules through targeted next-generation sequencing technology, exploring their predictive value for postoperative pathological malignancy, aiming to provide new non-invasive biomarkers for preoperative risk assessment of BI-RADS 4 lesions, assist clinical decision-making, reduce unnecessary surgical interventions, and has important clinical application prospects. The 49-gene panel evaluated in this study was selected based on three complementary principles: (1) established roles as driver genes in breast cancer, supported by large-scale genomic databases including TCGA, METABRIC, COSMIC, and ClinVar; (2) clinical actionability, including genes that are direct therapeutic targets (ERBB2, PIK3CA, ESR1) or inform treatment eligibility (BRCA1, BRCA2, ATM); and (3) comprehensive pathway coverage, encompassing DNA damage repair (BRCA1, BRCA2, ATM, CHEK2, PALB2), cell cycle regulation (CDK4, CDK6, CDKN2A, RB1), PI3K/AKT/mTOR signaling (PIK3CA, PTEN, AKT1/2/3, MTOR), hormone receptor signaling (ESR1, AR, FOXA1), receptor tyrosine kinase signaling (ERBB2, ERBB3, EGFR, FGFR1/2), and chromatin remodeling (ARID1A, KMT2C, KMT2D). Not all 49 genes are expected to contribute equally; genes with the highest mutational frequency and most established functional significance in breast cancer (BRCA1, TP53, ESR1, ERBB2) were prioritized for in-depth analysis, as detailed in Section 2.6 and the Results.

## 2 Materials and methods

### 2.1 Study subjects

This study retrospectively collected clinical data of patients with BI-RADS 4 breast nodules at our hospital from January 2020 to January 2024. Inclusion criteria: (1) female patients aged ≥18 years; (2) assessed as BI-RADS 4 (4A, 4B, or 4C) by breast ultrasound or mammography; (3) peripheral blood samples collected within 1 week before surgery; (4) underwent surgical treatment and obtained definitive pathological diagnosis; (5) complete clinical data. Exclusion criteria: (1) previous history of malignant tumors; (2) received neoadjuvant chemotherapy, radiotherapy, or endocrine therapy before surgery; (3) combined with other malignant tumors; (4) pregnant or lactating patients; (5) hematological or immunological diseases; (6) unqualified sample quality or missing data.

286 patients were initially screened, and 74 were excluded (21 with previous malignant tumor history, 18 with preoperative treatment, 9 with other tumors, 5 pregnant or lactating, 12 with hematological or immune system diseases, 9 with unqualified sample quality or missing data). Finally, 212 patients were included, including 128 cases in the benign group (60.4%) and 84 cases in the malignant group (39.6%), divided according to postoperative pathological results. This study was approved by the hospital ethics committee.

### 2.2 Clinical data collection

General patient data were retrospectively collected, including age, body mass index (BMI), menstrual status, family history, *etc.*,; imaging data included tumor size, location, BI-RADS classification (4A/4B/4C) [in this study, 92 cases (43.4%) were grade 4A, 78 cases (36.8%) were grade 4B, and 42 cases (19.8%) were grade 4C]; laboratory indicators included tumor markers (CA15-3, CEA, *etc.*). Malignant lesions were classified and graded according to the World Health Organization Classification of Breast Tumours, fifth edition, 2019.

### 2.3 Sample collection and processing

According to hospital standard operating procedures, all included patients had 10 mL of peripheral venous blood collected on an empty stomach in the morning within 1 week before surgery, placed in blood collection tubes containing EDTA anticoagulant. Within 2 h after blood collection, plasma was separated by centrifugation at 1,600 *g* for 10 min at 4 °C, then centrifuged at 16,000 *g* for 10 min at 4 °C to remove cell debris. The obtained plasma was stored at −80 °C for future use. In this study, the processing time of all samples met the requirements of standard operating procedures (time from blood collection to plasma separation <2 h), and all samples were tested within 6 months.

### 2.4 Peripheral blood cell-free DNA extraction

cfDNA was extracted from plasma using QIAamp Circulating Nucleic Acid Kit (Qiagen, Germany), strictly following the kit instructions. 2 mL plasma sample was taken, proteinase K and lysis buffer were added, incubated at 56 °C for 30 min, then purified and washed through adsorption column, and finally eluted with 50 μL elution buffer. cfDNA concentration was measured using Qubit 4.0 fluorometer (Thermo Fisher, USA), and DNA fragment distribution was assessed using Agilent 2,100 Bioanalyzer. Quality control standards: total cfDNA ≥10 ng (concentration ≥5 ng/μL), fragment size distribution main peak at 160–170 bp (corresponding to DNA fragments protected by mononucleosome), secondary peak at 320–340 bp (corresponding to dinucleosome fragments).

### 2.5 Library construction and targeted sequencing

Sequencing libraries were constructed using cfDNA library preparation kit (VAHTS Universal DNA Library Prep Kit, Vazyme, China). 10–20 ng cfDNA was used for end repair, A-tailing, adapter ligation, purification, and PCR amplification (9–10 cycles). Targeted capture of 49 breast cancer-related genes was performed using a custom-designed hybridization capture panel. Library quality control standards: library concentration ≥2 nM measured by Qubit 4.0, library main peak at 300–500 bp detected by Agilent 2,100, no obvious adapter dimer peak (<100 bp). Sequencing was performed using Illumina NovaSeq 6,000 platform, with mean on-target depth of 500–1,000×, paired-end sequencing mode (PE150), to ensure sufficient sensitivity for low-frequency variant detection.

### 2.6 Gene mutation analysis

Sequencing data underwent quality control using FastQC (v0.11.9), and adapter sequences and low-quality bases (Q < 20) were removed using Trimmomatic software (v0.39). High-quality reads were aligned to the human reference genome (hg38) using BWA-MEM algorithm (v0.7.17, parameters: M -t 8), sorted using SAMtools (v1.15), and deduplicated using Picard tools (v2.27.4) MarkDuplicates function.

Gene mutation analysis was performed using the following methods:

1. cfDNA concentration and fragment analysis: cfDNA fragment length was counted; short fragment ratio (SFR) was defined as: the ratio of <150 bp fragment count to [150 bp, 250 bp) fragment count, i.e., SFR = N (<150 bp)/N [150 bp, 250 bp).
2. Tumor Mutation Burden (TMB): Somatic mutations in the target gene panel were called using VarScan2 (v2.4.4) with a minimum variant allele frequency (VAF) threshold of 0.5%. TMB was calculated as the number of somatic non-synonymous mutations per megabase of sequenced target region (unit: mut/Mb). Only variants not present in germline databases (gnomAD, 1,000 Genomes) were included.
3. Mutation Allele Frequency (MAF): For each identified somatic variant, MAF was calculated as the ratio of variant reads to total reads at the locus. Genome-wide average MAF was computed for each sample as a summary statistic reflecting overall cfDNA tumor fraction. It should be noted that MAF as measured here reflects the allele frequency of somatic variants within the cfDNA pool, which includes contributions from both tumor-derived and non-tumor-derived DNA; this is distinct from the ctDNA fraction *per se*, which would require additional deconvolution.
4. Gene Mutation Score (GMS): A composite score integrating TMB, MAF, and mutation patterns across key driver gene categories was developed to summarize the overall pathogenic mutation burden at the sample level. The gene categories included were: tumor suppressors (BRCA1, BRCA2, TP53, PTEN), oncogenes (PIK3CA, ERBB2, AKT1), and hormone receptor pathway genes (ESR1, AR). These categories were selected because they represent the three major oncogenic axes in breast cancer—genomic integrity/apoptosis, proliferative signaling, and endocrine regulation—and have the highest frequency of actionable somatic mutations in breast cancer genomic studies. The formula is: GMS = Σ[wi × log2 (Mi/Ei)], where wi is the functional weight of gene category i assigned based on established breast cancer pathway importance in the literature (not empirically optimized in this dataset), Mi is the observed mutation coverage/frequency, and Ei is the expected background mutation rate from matched non-tumor references. GMS score ranges from −5 to +5; positive values indicate enriched pathogenic mutation burden. The GMS model does not currently apply differential weights to specific mutation types (e.g., transitions vs. transversions); somatic non-synonymous substitutions of both types were included uniformly. The biological significance of transition/transversion patterns (e.g., as signatures of APOBEC activity or oxidative damage) warrants exploration in future work.
5. Gene-specific mutation profiling: Focused analysis of all 49 breast cancer-related genes in the panel [including BRCA1, BRCA2, TP53, PIK3CA, PTEN, ESR1, ERBB2, CDH1, AKT1, GATA3, MAP3K1, TBX3, RUNX1, CBFB, MYC, CCND1, FGFR1, FGFR2, EGFR, MET, RB1, ATM, CHEK2, PALB2, CDK4, CDK6, CDKN2A, MDM2, NF1, ARID1A, ARID1B, KMT2C, KMT2D, NCOR1, FOXA1, RARA, AR, PGR, ERBB3, IGF1R, PIK3R1, AKT2, AKT3, MTOR, TSC1, TSC2, STK11, SMAD4, TGFBR2], including somatic mutation detection rate, VAF, and functional annotation (pathogenic/likely pathogenic classification based on ClinVar and COSMIC databases). This analysis differs from Method 4 in scope and purpose: Method 4 constructs a composite score (GMS) summarizing mutation burden across predefined gene category groups at the sample level, whereas Method 5 performs individual gene-level profiling across all 49 panel genes to characterize the mutation landscape in each patient. For the in-depth comparative analysis (Section 3.4), eight representative genes were selected from the full panel based on their high mutational frequency in this cohort and established clinical significance in breast cancer: BRCA1, TP53, ESR1, ERBB2 (showing significant between-group differences) and PIK3CA, PTEN, CDH1, GATA3 (included for completeness and comparison). Mutation types analyzed included somatic single-nucleotide variants (SNVs, both transitions and transversions) and small insertions/deletions (indels); copy number variants were not assessed in this study.

### 2.7 Statistical analysis

Statistical analysis was performed using SPSS 26.0 software and R language (version 4.2.1). For continuous variables, normality was first assessed using Shapiro-Wilk test and Q-Q plots. Data conforming to normal distribution were expressed as mean ± standard deviation (x̄±s), and independent sample t-test was used for comparison between two groups; data with non-normal distribution were expressed as median (interquartile range) [M(Q1,Q3)], and Mann-Whitney U test (Z value) was used for comparison between two groups. Categorical variables were expressed as number (percentage) [n (%)], and chi-square test or Fisher’s exact test was used. For multiple group comparisons, one-way analysis of variance (ANOVA) was used for normally distributed data, and Kruskal–Wallis H test was used for non-normally distributed data. When differences were statistically significant, Bonferroni method was used for pairwise comparisons.

Correlation analysis between gene mutation indicators and clinicopathological parameters was performed using Spearman rank correlation analysis, calculating correlation coefficient r and its P value. For multiple hypothesis testing, Bonferroni method was used for correction, with corrected significance level α' = 0.05/number of tests. Univariate and multivariate logistic regression analysis was used to analyze factors affecting postoperative pathological malignancy. Variables with P < 0.05 in univariate analysis were included in multivariate analysis, using backward stepwise method (removal criterion P > 0.10), calculating odds ratio (OR) and its 95% confidence interval (95%CI).

Receiver operating characteristic (ROC) curves were plotted to evaluate the predictive efficacy of various indicators and combined models, calculating area under the curve (AUC) and its 95%CI. The optimal cutoff value was determined according to the principle of maximizing Youden index, and corresponding sensitivity, specificity, positive predictive value (PPV), and negative predictive value (NPV) were calculated. DeLong test was used to compare differences in AUC between different models. For the combined model, Bootstrap method (1,000 repeated samplings) was used for internal validation, calculating corrected AUC. Two-tailed test, P < 0.05 was considered statistically significant.

## 3 Results

### 3.1 Comparison of clinicopathological characteristics between two groups

There were statistically significant differences between the two groups in age, BI-RADS classification, tumor size, CA15-3, and CEA levels (P < 0.05), while there were no statistically significant differences in BMI, menstrual status, family history, and tumor location (P > 0.05) (Table 1).

**TABLE 1:** Comparison of clinicopathological characteristics between two groups.

Indicator	Benign group (n = 128)	Malignant group (n = 84)	Statistics	P value
Age (years, x̄±s)	45.3 ± 11.2	52.6 ± 10.8	t = 4.712	<0.001
BMI (kg/m^2^, x̄±s)	23.6 ± 3.1	24.1 ± 3.4	t = 1.125	0.262
Menstrual status [n (%)]	​	​	χ^2^ = 2.847	0.092
Premenopausal	78 (60.9)	42 (50.0)	​	​
Postmenopausal	50 (39.1)	42 (50.0)	​	​
Family history [n (%)]	18 (14.1)	16 (19.0)	χ^2^ = 1.125	0.289
Tumor location [n (%)]	​	​	χ^2^ = 3.658	0.454
Upper outer quadrant	52 (40.6)	38 (45.2)	​	​
Upper inner quadrant	28 (21.9)	16 (19.0)	​	​
Lower outer quadrant	24 (18.8)	14 (16.7)	​	​
Lower inner quadrant	16 (12.5)	10 (11.9)	​	​
Central region	8 (6.3)	6 (7.1)	​	​
Tumor size (cm, x̄±s)	1.8 ± 0.7	2.4 ± 0.9	t = 5.376	<0.001
BI-RADS classification [n (%)]	​	​	χ^2^ = 22.458	<0.001
4A	68 (53.1)	24 (28.6)	​	​
4B	46 (35.9)	32 (38.1)	​	​
4C	14 (10.9)	28 (33.3)	​	​
CA15-3 (U/mL) [M(Q1,Q3)]	18.2 (12.5,24.6)	25.8 (18.3,35.4)	Z = −4.892	<0.001
CEA (ng/mL) [M(Q1,Q3)]	2.1 (1.3,3.2)	3.6 (2.2,5.8)	Z = −5.124	<0.001

Among 84 malignant cases, pathological classification according to WHO Classification of Breast Tumours fifth edition: invasive ductal carcinoma 66 cases (78.6%), invasive lobular carcinoma 10 cases (11.9%), mixed carcinoma 4 cases (4.8%), other special types 4 cases (4.8%, including mucinous carcinoma 2 cases, medullary carcinoma 1 case, metaplastic carcinoma 1 case). Histological grade distribution: G1 grade 18 cases (21.4%), G2 grade 42 cases (50.0%), G3 grade 24 cases (28.6%) (Table 2). Regarding immunohistochemical profiles, complete data were available for 72 of 84 malignant cases: estrogen receptor (ER) positivity was detected in 48 cases (66.7%), progesterone receptor (PR) positivity in 42 cases (58.3%), and HER2 overexpression (IHC 3+ or FISH-amplified) in 18 cases (25.0%). Ki-67 proliferation index was ≥20% in 36 cases (50.0%), indicative of high proliferative activity. Based on surrogate molecular subtyping, the distribution was as follows: Luminal A (ER+ and/or PR+, HER2−, Ki-67 < 20%) in 24 cases (33.3%), Luminal B (ER+ and/or PR+, HER2− with Ki-67 ≥ 20%, or HER2+) in 24 cases (33.3%), HER2-enriched (ER−, PR−, HER2+) in 6 cases (8.3%), and Triple-negative (ER−, PR−, HER2−) in 18 cases (25.0%). Lymph node status at surgery was available for all 84 malignant cases: lymph node-negative in 54 cases (64.3%) and lymph node-positive in 30 cases (35.7%). TNM staging showed Stage I in 30 cases (35.7%), Stage II in 42 cases (50.0%), and Stage III in 12 cases (14.3%). Incomplete immunohistochemical data for 12 cases is acknowledged as a limitation of the retrospective study design.

**TABLE 2:** Distribution of pathological classification and grading in malignant cases.

Pathological features	Number (n = 84)	Percentage (%)
Pathological classification		
Invasive ductal carcinoma	66	78.6
Invasive lobular carcinoma	10	11.9
Mixed carcinoma	4	4.8
Other special types	4	4.8
Histological grade		
G1 grade	18	21.4
G2 grade	42	50.0
G3 grade	24	28.6

### 3.2 Comparison of cfDNA basic characteristics between two groups

The cfDNA concentration in the malignant group was higher than that in the benign group (P < 0.001). In terms of fragment length distribution, the proportion of short fragments (<120 bp) and SFR in the malignant group were higher than those in the benign group (P < 0.001), and the proportion of mononucleosome fragments (120–180 bp) was lower than that in the benign group (P < 0.001). There was no statistically significant difference in the proportion of dinucleosome and polynucleosome fragments between the two groups (P > 0.05). TMB in the malignant group was significantly higher than that in the benign group (P < 0.001) (Table 3).

**TABLE 3:** Comparison of cfDNA Basic Characteristics and TMB Between Two Groups.

Indicator	Benign group (n = 128)	Malignant group (n = 84)	Statistics	P value
cfDNA concentration (ng/mL, x̄±s)	8.6 ± 3.2	14.3 ± 5.8	t = 8.426	<0.001
Short fragment proportion (%) (a) [M(Q1,Q3)]	12.4 (9.2,15.8)	18.7 (14.3,24.5)	Z = −6.745	<0.001
Mononucleosome fragment proportion (%, x̄±s)	62.8 ± 8.4	56.2 ± 9.7	t = −5.236	<0.001
Dinucleosome fragment proportion (%, x̄±s)	21.5 ± 5.2	20.8 ± 6.1	t = −0.876	0.382
Polynucleosome fragment proportion (%) [M(Q1,Q3)]	3.2 (2.1,4.8)	3.6 (2.3,5.2)	Z = −1.234	0.217
SFR(b) [M(Q1,Q3)]	0.78 (0.62,0.95)	1.24 (0.96,1.58)	Z = −7.892	<0.001
TMB (mut/Mb) [M(Q1,Q3)]	0.39 (0.27,0.68)	1.31 (0.99,1.63)	Z = −10.406	<0.001

(a) Short fragment proportion refers to the percentage of cfDNA, with fragment length <120 bp; (b) SFR (short fragment ratio) is defined as the ratio of fragment count <150 bp to fragment count [150 bp, 250 bp).

### 3.3 Comparison of gene mutation features between two groups

The overall mutation detection rate in the malignant group was significantly lower than in the benign group (P < 0.001). In different gene categories, mutation rates in tumor suppressor genes (BRCA1, TP53, PTEN) and oncogenes (ERBB2, PIK3CA) were higher in the malignant group compared to benign group (P < 0.001), while no statistically significant difference was found in the mutation rate of variant-of-unknown-significance (VUS) variants between the two groups (P > 0.05). The GMS in the malignant group was significantly lower than in the benign group (P < 0.001) (Table 4).

**TABLE 4:** Comparison of gene mutation features between two groups.

Indicator	Benign group (n = 128)	Malignant group (n = 84)	Statistics	P value
Overall mutation detection rate (%, x̄±s)	2.84 ± 0.52	2.18 ± 0.63	t = −8.142	<0.001
Pathogenic/likely pathogenic variant rate (RPK, x̄±s)	45.6 ± 8.3	36.2 ± 9.5	t = −7.524	<0.001
VUS variant rate (RPK, x̄±s)	38.2 ± 6.7	36.8 ± 7.2	t = −1.432	0.154
Mutation allele frequency—driver genes (RPK) [M(Q1,Q3)]	42.5 (35.2,51.8)	32.6 (26.4,40.3)	Z = −6.235	<0.001
GMS (x̄±s)	1.82 ± 0.68	0.95 ± 0.74	t = −8.756	<0.001

### 3.4 Analysis of gene mutation features in breast cancer driver genes

Based on reported importance of breast cancer driver genes, clinical application value, and preliminary screening results in this study, 8 representative breast cancer-related genes (BRCA1, TP53, ESR1, ERBB2, PIK3CA, PTEN, CDH1, GATA3) were selected for in-depth analysis. Mutation detection rates and pathogenic variant scores in BRCA1, TP53, ESR1, and ERBB2 in the malignant group were significantly lower than those in the benign group (P < 0.05), while there was no statistically significant difference in PIK3CA, PTEN, CDH1, and GATA3 between the two groups (P > 0.05). Bonferroni correction was applied to 16 hypothesis tests for 8 genes (corrected α = 0.05/16 = 0.0031), and differences in mutation detection rates and pathogenic variant scores of BRCA1, TP53, ESR1, and ERBB2 remained statistically significant (P < 0.0031) (Table 5).

**TABLE 5:** Comparison of gene mutation features in representative breast cancer driver genes.

Gene	Mutation Detection Rate (RPK, x̄±s) Benign group	Mutation Detection Rate (RPK, x̄±s) Malignant group	Statistics	P value	Pathogenic Variant Score (x̄±s) Benign group	Pathogenic Variant Score (x̄±s) Malignant group	Statistics	P value
BRCA1	48.3 ± 9.2	38.6 ± 10.5	t = −6.987	<0.001	1.96 ± 0.62	1.15 ± 0.71	t = −8.524	<0.001
TP53	52.6 ± 10.1	41.2 ± 11.8	t = −7.342	<0.001	2.14 ± 0.58	1.28 ± 0.68	t = −9.456	<0.001
ESR1	46.8 ± 8.7	37.5 ± 9.6	t = −7.128	<0.001	1.88 ± 0.65	1.06 ± 0.72	t = −8.326	<0.001
ERBB2	44.2 ± 9.5	35.8 ± 10.2	t = −5.987	<0.001	1.75 ± 0.69	0.98 ± 0.76	t = −7.445	<0.001
PIK3CA	42.5 ± 8.9	40.2 ± 9.3	t = −1.784	0.076	1.62 ± 0.71	1.42 ± 0.78	t = −1.896	0.059
PTEN	40.8 ± 7.6	38.9 ± 8.4	t = −1.672	0.096	1.54 ± 0.68	1.38 ± 0.73	t = −1.587	0.114
CDH1	39.6 ± 8.2	37.8 ± 8.9	t = −1.485	0.139	1.48 ± 0.72	1.31 ± 0.76	t = −1.623	0.106
GATA3	41.3 ± 7.9	39.5 ± 8.6	t = −1.542	0.125	1.58 ± 0.69	1.44 ± 0.74	t = −1.389	0.166

After Bonferroni multiple comparison correction, differences in BRCA1, TP53, ESR1, and ERBB2 remain statistically significant (P < 0.0031).

#### 3.4.1 Differences in gene mutation features among different pathological classifications and grades

The relationship between different pathological classifications, histological grades, and gene mutation indicators in malignant cases was further analyzed. Due to the small sample size of mixed carcinoma (4 cases) and other special types (4 cases), pathological classification comparison only included invasive ductal carcinoma (66 cases) and invasive lobular carcinoma (10 cases). There was no statistically significant difference in TMB and GMS between invasive ductal carcinoma and invasive lobular carcinoma patients (P > 0.05), but the large difference in sample sizes between the two groups resulted in insufficient statistical test power (actual test power about 30%, requiring at least 25 cases/group to achieve 80% power). Results should be interpreted cautiously and cannot rule out the possibility of actual differences. Comparison of gene mutation indicators among different histological grades showed that TMB differed among G1, G2, and G3 tumors (H = 7.144, P = 0.028), with a trend toward higher TMB in G3 tumors. Overall mutation detection rate and driver gene MAF were lower in G3 tumors than in lower-grade tumors, while GMS was lower in G2/G3 tumors than in G1 tumors (Table 6).

**TABLE 6:** Comparison of gene mutation indicators among different pathological features in malignant cases.

Feature	n	TMB [M(Q1,Q3)]	GMS (x̄±s)	Overall mutation rate (x̄±s)	Driver gene MAF (RPK, x̄±s)
Pathological classification(a)					
Invasive ductal carcinoma	66	1.31(0.99,1.62)	0.93 ± 0.76	2.16 ± 0.65	36.0 ± 9.8
Invasive lobular carcinoma	10	1.24(0.96,1.76)	1.02 ± 0.68	2.25 ± 0.58	37.2 ± 8.6
P value	​	0.993	0.715	0.682	0.728
Histological grade					
G1 grade	18	0.98(0.74,1.59)	1.28 ± 0.68(b)	2.45 ± 0.58(b)	40.5 ± 8.9(b)
G2 grade	42	1.31(1.07,1.55)	1.02 ± 0.71(b)	2.24 ± 0.61(b)	37.1 ± 9.2(b)
G3 grade	24	1.51(1.24,1.77)	0.62 ± 0.72	1.86 ± 0.62	32.4 ± 9.6
H/F Value	​	H = 7.144	F = 6.892	F = 5.784	F = 5.326
P value	​	0.028	0.002	0.005	0.007

(a) Mixed carcinoma and other special types were not included in comparison due to small sample size (4 cases each); comparison of invasive ductal carcinoma vs. invasive lobular carcinoma has insufficient statistical test power due to large difference in sample size (66 cases vs. 10 cases, about 30%), negative results should be interpreted cautiously and cannot rule out the possibility of actual differences; (b) Compared with G3 grade, P < 0.05; comparison between G1 and G2 grades, P > 0.05.

### 3.5 Correlation analysis between gene mutation indicators and clinicopathological parameters

Spearman correlation analysis showed that TMB was positively correlated with tumor size (r = 0.412), BI-RADS classification (r = 0.386), CA15-3 (r = 0.358), CEA (r = 0.341), and histological grade (r = 0.338) (P < 0.001), and negatively correlated with overall mutation detection rate (r = −0.524) and GMS (r = −0.498) (P < 0.001). GMS was negatively correlated with BI-RADS classification (r = −0.425), tumor size (r = −0.392), and histological grade (r = −0.356) (P < 0.001). Considering multiple comparison issues, Bonferroni correction was applied to 30 independent correlation tests (α = 0.05/30 ≈ 0.0017), and the above main correlations remained statistically significant (P < 0.0017) (Table 7).

**TABLE 7:** Spearman correlation analysis between gene mutation indicators and clinicopathological parameters.

Indicator	TMB	Overall mutation detection rate	Driver gene MAF	GMS
Age	0.245**	−0.198**	−0.156*	−0.187**
Tumor size	0.412***	−0.367***	−0.328***	−0.392***
BI-RADS classification	0.386***	−0.358***	−0.342***	−0.425***
CA15-3	0.358***	−0.312***	−0.289***	−0.334***
CEA	0.341***	−0.298***	−0.276***	−0.318***
Histological grade(a)	0.338***	−0.324***	−0.298***	−0.356***
Overall mutation detection rate	−0.524***	1	0.612***	0.658***
Driver gene MAF	−0.468***	0.612***	1	0.584***
GMS	−0.498***	0.658***	0.584***	1

*P < 0.05, **P < 0.01, ***P < 0.001; (a) Analysis performed only for 84 malignant cases; after Bonferroni correction (α = 0.0017), marked correlations remain statistically significant.

### 3.6 Analysis of factors affecting postoperative pathological malignancy

Univariate analysis showed that age, tumor size, BI-RADS classification, CA15-3, CEA, cfDNA concentration, TMB, overall mutation detection rate, driver gene MAF, BRCA1/TP53 mutation status, and GMS were associated with postoperative pathological malignancy (P < 0.05). The above variables were included in multivariate logistic regression analysis. Results showed that age (OR = 1.058), BI-RADS classification (OR = 2.874), TMB (OR = 4.326), and GMS (OR = 0.178) were independent predictors of postoperative pathological malignancy in BI-RADS 4 breast nodules (P < 0.05) (Table 8).

**TABLE 8:** Multivariate logistic regression analysis of postoperative pathological malignancy in BI-RADS 4 breast nodules.

Variable	β	SE	Wald χ2	P value	OR	95%CI
Age	0.056	0.018	9.654	0.002	1.058	1.021–1.096
BI-RADS classification	1.056	0.285	13.745	<0.001	2.874	1.643–5.027
TMB	1.465	0.342	18.356	<0.001	4.326	2.214–8.452
GMS	−1.726	0.398	18.824	<0.001	0.178	0.082–0.388
Constant	−3.124	1.256	6.187	0.013	0.044	​

Included variables include age, tumor size, BI-RADS, classification, CA15-3, CEA, cfDNA, concentration; TMB, overall mutation detection rate, driver gene MAF, BRCA1/TP53 mutation status, GMS; backward stepwise method was used, retaining variables with P < 0.05.

### 3.7 Efficacy evaluation of prediction models

ROC curve analysis showed that TMB had an AUC of 0.824 (95%CI: 0.765–0.883) for predicting postoperative pathological malignancy, with sensitivity of 78.6% and specificity of 79.7%; GMS had an AUC of 0.852 (95%CI: 0.798–0.906), with sensitivity of 81.0% and specificity of 82.8%. The combined model (age + BI-RADS classification + TMB + GMS) had an AUC of 0.923 (95%CI: 0.884–0.962), sensitivity of 88.1%, specificity of 89.1%, PPV of 84.1%, and NPV of 91.8%. The AUC of the combined model was higher than that of single indicators (P < 0.001). Bootstrap internal validation showed that the corrected AUC of the combined model was 0.918 (95%CI: 0.876–0.957) (Table 9).

**TABLE 9:** Comparison of efficacy of different indicators and combined models for predicting postoperative pathological malignancy.

Model	AUC	95%CI	Cutoff value	Sensitivity (%)	Specificity (%)	PPV (%)	NPV (%)
Age	0.682	0.612–0.752	49.5 years	66.7	64.1	55.4	74.2
BI-RADS classification	0.714	0.646–0.782	≥4B	71.4	64.8	57.1	77.4
cfDNA concentration	0.765	0.702–0.828	11.2 ng/mL	73.8	71.9	63.3	80.7
TMB	0.824	0.765–0.883	1.05 mut/Mb	78.6	79.7	71.0	85.4
Overall mutation detection rate	0.798	0.737–0.859	2.45%	76.2	75.8	67.4	82.9
Driver gene MAF	0.782	0.720–0.844	40.5 RPK	75.0	73.4	65.6	81.5
GMS	0.852	0.798–0.906	1.32	81.0	82.8	75.6	86.9
Combined model	0.923	0.884–0.962	0.52(a)	88.1	89.1	84.1	91.8

(a) The cutoff value for the combined model represents the predicted probability threshold, determined by maximizing the Youden index; the combined model includes. For age, BI‐RADS classification, cfDNA concentration, TMB, and the combined model, values above the cutoff indicate higher malignancy risk. For overall mutation detection rate, driver gene MAF, and GMS, values below the cutoff indicate higher malignancy risk.

## 4 Discussion

This study found that malignant patients showed typical cfDNA alteration patterns, manifested as elevated tumor mutation burden and decreased detection rates of selected driver-gene mutation-related indicators. This finding is consistent with previous studies (Ungerer et al., 2022) and with recent work demonstrating that cfDNA fragmentation and mutational landscapes reflect the underlying genomic instability of breast tumors. The mutational landscape of cfDNA is shaped by the genetic instability and clonal evolution of tumor cells. Due to the accumulation of somatic mutations in oncogenes and tumor suppressor genes, enhanced activation of apoptotic pathways, and altered DNA repair mechanisms, tumor cells release cfDNA with unique mutational signatures (Zhu et al., 2023). Notably, the elevated TMB observed in malignant cases in this study is consistent with the concept of genomic instability as a hallmark of cancer, particularly in higher-grade tumors where defective homologous recombination repair—driven by BRCA1/2 and TP53 dysfunction—leads to accelerated somatic mutation accumulation (An et al., 2023). The increase in TMB may reflect heightened genomic instability during tumor cell apoptosis and necrosis, leading to the shedding of mutation-bearing DNA fragments into the circulation. In this study, the median TMB of the malignant group was 1.31 mut/Mb, significantly higher than 0.39 mut/Mb in the benign group, and this indicator was positively correlated with tumor grade (r = 0.338), suggesting that TMB can serve as a potential marker reflecting tumor biological behavior. This aligns with findings from Barbirou et al. (2022), who demonstrated that cfDNA-based molecular profiling could differentiate malignant from benign breast lesions, supporting the translational relevance of our approach in the specific context of BI-RADS 4 lesions.

More importantly, this study found that malignant patients showed significant gene-level alterations, manifested as decreased overall mutation detection rates in regulatory contexts, reduced driver gene MAF in certain tumor suppressor categories, and a lower composite Gene Mutation Score. The biological basis for these changes lies in the complex interplay between driver gene mutations and epigenetic silencing in tumor cells (Zhang and Li, 2022). In normal cells, tumor suppressor genes such as BRCA1 and TP53 maintain genome integrity through DNA repair and apoptosis pathways. This ordered regulation is crucial for cell homeostasis (Oyarzún-Cisterna et al., 2024). However, in breast cancer cells, widespread genetic and epigenetic changes lead to disruption of these pathways, ultimately reflected in the mutational patterns detectable in cfDNA (Sadida et al., 2024). The apparently paradoxical observation that the GMS—which integrates pathogenic mutation burden—is lower in the malignant group warrants biological explanation. We hypothesize that this reflects a shift in mutational architecture in malignant tumors: while TMB (overall non-synonymous mutation count) increases, the proportion of mutations falling in the specific regulatory and driver gene regions captured by the GMS formula (which emphasizes expected vs. observed mutation distribution) may be diluted by widespread background genomic instability. This is consistent with the concept that cancer genomes accumulate many passenger mutations alongside fewer functionally dominant driver events. In this study, the GMS in the malignant group decreased by 47.8% compared to the benign group, reflecting profound changes in the genomic landscape of breast cancer cells and supporting GMS as a complementary—rather than redundant—predictor to TMB.

This study focused on analyzing mutation patterns in promoter/regulatory regions of breast cancer driver genes, and found that key genes such as BRCA1, TP53, ESR1, and ERBB2 showed significantly altered mutation detection rates and pathogenic variant scores in malignant cases, with differences remaining statistically significant even after rigorous multiple comparison correction. This finding has important biological and clinical significance. As important tumor suppressor genes, pathogenic mutations in BRCA1 and TP53 are closely related to dysfunctional DNA damage repair in breast cancer development (Xu et al., 2024). Studies have shown that the type and frequency of mutations in driver gene coding and regulatory regions affect transcription factor binding and gene expression activity (Sahrhage et al., 2024). In this study, the mutation detection rate in the BRCA1 regulatory region was lower in the malignant group than in the benign group. which may reflect abnormal regulation of this gene’s expression, closely related to DNA damage repair dysfunction in breast cancer (Arun et al., 2024).

ESR1 and ERBB2 are important therapeutic targets and prognostic markers for breast cancer. Changes in mutation features in their regulatory regions may be related to tumor hormone receptor status and HER2 expression levels (Li et al., 2022). Previous studies mainly focused on coding mutations and copy number variations of these genes. This study reveals new evidence of their regulatory abnormalities from a genomic perspective (Albrecht et al., 2025). Notably, mutation features of genes such as PIK3CA and PTEN showed no statistically significant differences between the two groups. This may be related to different modes of action of these genes in breast cancer. PIK3CA mainly activates the PI3K signaling pathway through hotspot coding mutations rather than regulatory region alterations (Chao et al., 2023). This suggests heterogeneity in mutational alteration patterns of different genes in tumorigenesis, requiring further research for clarification.

This study found that among malignant cases, histological G3 grade patients had significantly higher TMB than G1 and G2 grades, while GMS, overall mutation rate, and driver gene MAF were significantly different from lower-grade tumors. This finding is consistent with the degree of genomic instability related to tumor differentiation (Iglesia et al., 2024). High-grade tumors have stronger proliferative activity and more significant genomic instability, with more severe driver gene mutation accumulation, leading to higher TMB and more complex mutational landscapes (Castellanos et al., 2023). Spearman correlation analysis showed that TMB was positively correlated with histological grade (r = 0.338), and GMS was negatively correlated with histological grade (r = −0.356), further supporting that gene mutation features can serve as molecular markers of tumor malignancy.

However, in this study, there was no statistically significant difference in gene mutation indicators between invasive ductal carcinoma and invasive lobular carcinoma, which may be related to sample size imbalance (66 cases vs. 10 cases), resulting in insufficient statistical test power. Previous studies have shown differences in molecular characteristics among different pathological types of breast cancer (Schwartz et al., 2023), but whether there are type-specific changes at the cfDNA gene mutation level requires further validation with larger sample sizes. In addition, there was no statistically significant difference in gene mutation indicators between G1 and G2 grades, suggesting that mutation burden may undergo more significant changes at specific stages of tumor progression (such as transformation to high grade) (Vanderstichele et al., 2022).

The combined prediction model established in this study integrated age, BI-RADS classification, TMB, and GMS, showing excellent predictive efficacy (AUC = 0.923, sensitivity 88.1%, specificity 89.1%). Compared with single indicators, the AUC of the combined model significantly improved, reflecting the advantage of multidimensional information integration (Sammut et al., 2022). Traditional clinical and imaging indicators, although having certain predictive value, have limited ability to distinguish BI-RADS 4 lesions with high heterogeneity. In this study, the AUC of BI-RADS classification was only 0.714, but after adding gene mutation profiling features, model performance greatly improved, providing more precise tools for clinical decision-making (Bai et al., 2025).

From a clinical application perspective, the prediction model established in this study has the following advantages: First, non-invasiveness, requiring only 10 mL of peripheral blood for testing, with high patient acceptance; Second, objectivity, based on quantitative genomic indicators, avoiding subjectivity in imaging assessment; Third, comprehensiveness, integrating information from multiple dimensions, better reflecting genetic characteristics of lesions (Wang et al., 2024). The negative predictive value of this model reaches 91.8%, suggesting that for patients predicted to be at low risk, close follow-up rather than immediate surgery can be considered, thereby reducing overtreatment. The positive predictive value is 84.1%, suggesting that for patients predicted to be at high risk, active surgical intervention or biopsy should be performed. This risk stratification strategy helps achieve individualized precision management (Chen et al., 2023).

In addition, cfDNA gene mutation analysis has potential for dynamic monitoring. Previous studies have shown that cfDNA mutational characteristics can reflect tumor burden and treatment response (Zaikova et al., 2024). For breast cancer patients receiving neoadjuvant therapy, monitoring changes in cfDNA gene mutation features may help early assessment of efficacy and prediction of prognosis. Although this study did not involve treatment monitoring, it lays a foundation for subsequent related research.

This study has the following limitations: First, this is a single-center retrospective study with relatively limited sample size and lack of external validation cohort. Although internal validation was performed using Bootstrap method, with corrected AUC still reaching 0.918, the generalizability and stability of the model require further validation through multicenter, large-sample prospective studies (Chang et al., 2025). Second, this study failed to obtain complete immunohistochemical information (such as ER, PR, HER2 status, Ki-67 index) for all malignant cases, limiting in-depth analysis of differences in gene mutation features among different molecular subtypes of breast cancer. Third, this study used targeted panel sequencing (500–1,000×), which is sufficient for somatic variant detection but has limited sensitivity for detecting low-frequency mutations outside the panel. Fourth, bioinformatics methods for mutation calling from cfDNA are not yet fully standardized, and different algorithms and parameters used in different studies may affect result comparability. Fifth, this study only analyzed cfDNA mutation features at a single preoperative time point, failing to dynamically monitor changes in mutation patterns, limiting understanding of their dynamic evolution.

Based on the findings and limitations of this study, future research can be deepened in the following directions: First, conduct multicenter prospective validation studies, expand sample size, especially increase the number of cases with different pathological types and molecular subtypes, to improve model generalizability and clinical practicality. Second, combine immunohistochemical and molecular typing information to explore differences in gene mutation features among different breast cancer subtypes and their biological significance. Third, integrate multi-omics information such as cfDNA methylation, copy number variations, and transcriptomic data to construct more comprehensive liquid biopsy prediction models. Fourth, conduct longitudinal studies to dynamically monitor changes in cfDNA gene mutation features during treatment, exploring their value in efficacy assessment and prognosis prediction. Fifth, deeply study molecular mechanisms underlying driver gene mutations in breast cancer, especially the roles of DNA repair pathways, oncogene amplification, and epigenetic modifiers, providing theoretical basis for targeted intervention. Sixth, optimize and standardize bioinformatics workflows for cfDNA mutation analysis, establish unified quality control standards and reference ranges, promoting clinical translation of this technology. Seventh, explore combined application of cfDNA gene mutation analysis with other liquid biopsy technologies (such as circulating tumor cells, exosomes, circulating tumor RNA) to construct multidimensional non-invasive diagnostic systems.

In summary, this study confirms that cfDNA breast cancer gene mutation profiling has good predictive value for postoperative pathological malignancy in BI-RADS 4 breast nodules, particularly TMB and GMS can serve as independent predictors. The prediction model combining clinical and imaging indicators shows excellent performance, providing a new non-invasive method for preoperative risk assessment of BI-RADS 4 lesions, helping achieve precise individualized clinical management. Although further validation and optimization are still needed, this study opens a new direction for the application of liquid biopsy in early breast cancer diagnosis and has important clinical translation prospects.

## Data Availability

The original contributions presented in the study are publicly available. This data can be accessed via the following link: https://figshare.com/s/bc633a7bc9be31afad80.
